# Computing Value from Quality and Quantity in Human Decision-Making

**DOI:** 10.1523/JNEUROSCI.0706-18.2018

**Published:** 2019-01-02

**Authors:** Archy O. de Berker, Zeb Kurth-Nelson, Robb B. Rutledge, Sven Bestmann, Raymond J. Dolan

**Affiliations:** ^1^Wellcome Trust Centre for Neuroimaging, University College London, London WC1N 3BG, United Kingdom,; ^2^Max Planck University College London Centre for Computational Psychiatry and Ageing Research, London WC1B 5EH, United Kingdom,; ^3^Sobell Department of Motor Neuroscience and Movement Disorders, University College London, London WC1N 3BG, United Kingdom,; ^4^Element AI, Montreal, Quebec H2W 2R2, Canada, and; ^5^DeepMind, London EC4 3TW, United Kingdom

**Keywords:** decision, number, value

## Abstract

How organisms learn the value of single stimuli through experience is well described. In many decisions, however, value estimates are computed “on the fly” by combining multiple stimulus attributes. The neural basis of this computation is poorly understood. Here we explore a common scenario in which decision-makers must combine information about quality and quantity to determine the best option. Using fMRI, we examined the neural representation of quality, quantity, and their integration into an integrated subjective value signal in humans of both genders. We found that activity within inferior frontal gyrus (IFG) correlated with offer quality, while activity in the intraparietal sulcus (IPS) specifically correlated with offer quantity. Several brain regions, including the anterior cingulate cortex (ACC), were sensitive to an interaction of quality and quantity. However, the ACC was uniquely activated by quality, quantity, and their interaction, suggesting that this region provides a substrate for flexible computation of value from both quality and quantity. Furthermore, ACC signals across subjects correlated with the strength of quality and quantity signals in IFG and IPS, respectively. ACC tracking of subjective value also correlated with choice predictability. Finally, activity in the ACC was elevated for choice trials, suggesting that ACC provides a nexus for the computation of subjective value in multiattribute decision-making.

**SIGNIFICANCE STATEMENT** Would you prefer three apples or two oranges? Many choices we make each day require us to weigh up the quality and quantity of different outcomes. Using fMRI, we show that option quality is selectively represented in the inferior frontal gyrus, while option quantity correlates with areas of the intraparietal sulcus that have previously been associated with numerical processing. We show that information about the two is integrated into a value signal in the anterior cingulate cortex, and the fidelity of this integration predicts choice predictability. Our results demonstrate how on-the-fly value estimates are computed from multiple attributes in human value-based decision-making.

## Introduction

Convergent evidence from human fMRI (O'Doherty et al., 2001; Montague and Berns, 2002; Kable and Glimcher, 2007; Knutson et al., 2007; FitzGerald et al., 2009; Levy et al., 2011) and nonhuman primate recordings (Schultz et al., 1997; Padoa-Schioppa and Assad, 2006; Hayden et al., 2011; Kennerley et al., 2011; Padoa-Schioppa and Schoenbaum, 2015) suggest that neural representations of subjective value are present in a wide variety of brain areas, potentially represented in an automatic fashion invariant to the task at hand (Lebreton et al., 2009; Grueschow et al., 2015). These value estimates are thought to provide input to value-comparison mechanisms to enable an appropriate decision between options (Padoa-Schioppa and Assad, 2006, 2008; Padoa-Schioppa, 2011; Xie and Padoa-Schioppa, 2016), a process variously characterized as evidence accumulation (Krajbich et al., 2010; De Martino et al., 2013; Polanía et al., 2014) or mutually inhibitory competition (Wang, 2008; Hunt et al., 2012; Chau et al., 2014). Representations of stimulus value also play a crucial role in reinforcement learning, where discrepancies between experienced and expected values give rise to the prediction errors that drive learning (Schultz et al., 1997; Sutton and Barto, 1998; Pessiglione et al., 2006; Rutledge et al., 2010; Kahnt et al., 2011).

Despite abundant and consistent evidence for value representations in specific brain areas, we still know little about how they come about and are integrated across multiple attributes. Efforts to isolate value signals within a neuroeconomic framework have used carefully controlled stimulus characteristics and action requirements in an effort to disambiguate value from its components (O'Doherty, 2014; Hunt et al., 2015). However, in the real world, we often need to construct valuations of never-before-seen objects. Recent studies using foraging tasks have emphasized that a more ethological contextualization of decision-making provides a richer account of the computations which underlying choice (Cisek and Kalaska, 2010; Kahnt et al., 2011; Chau et al., 2014; Kolling et al., 2014), highlighting a need to understand the individual component processes that contribute to value estimation. In this experiment, we drew inspiration from such foraging tasks to ask how current option value is constructed from two component parts, quality and quantity.

We designed an experiment where participants integrated information about the quality of a giftcard (how subjectively valuable it was for them to be able to spend money at a particular store) and its quantity (how much money was on the giftcard). In a behavioral session, we characterized the combination of quality and quantity to form integrated values using an auction procedure [Becker-DeGroot-Marschak (BDM); Becker et al., 1964], allowing us to select giftcards with distinct qualities. In the subsequent fMRI experiment, participants evaluated a series of individual giftcards without any choice requirement, allowing us to examine correlates of quality, quantity, and value that were uncontaminated by decision-related signals (Hunt et al., 2015).

We found that quality was represented in the inferior frontal gyrus (IFG), extending into the lateral prefrontal cortex (PFC). Conversely, quantity was associated with increasing activity in the bilateral intraparietal sulcus (IPS). To identify regions in which the two might be interacting in a manner consistent with the calculation of value, we formulated an explicit interaction term. This interaction term captures the fact that an extra unit of money on the highest quality giftcard is more valuable to the subject than an extra unit on the low-quality giftcard; you would rather have another dollar to spend at a shop you really like than at one you dislike. This interaction (higher slope of quantity coding with higher quality) correlated with activity in the posterior cingulate cortex and bilateral superior temporal regions. Anterior cingulate cortex (ACC) displayed a conjunction of all three effects, indicative of a substrate for the calculation of integrated subjective value from its component parts. In keeping with this, we also observed repetition suppression (RS) for integrated subjective value in the cingulate cortex, with activity covarying with the absolute difference in value between stimuli presented in consecutive trials.

## Materials and Methods

### Participants

Forty-seven participants (25 males) participated in the behavioral study, with 26 returning for an fMRI session. Of these, one participant failed to complete the experiment due to ill health, leaving 25 participants in total for the imaging study. Both studies were approved by a local ethics committee (Research Ethics Committee UCL, reference 3450/002). Based on pilot experiments, we selected 13 giftcards that were well known to the participant population, maximized between-subject variability, and displayed minimal correlations between cards (i.e., preferences for a given card could not be predicted from preferences for other cards).

**Figure 1.**
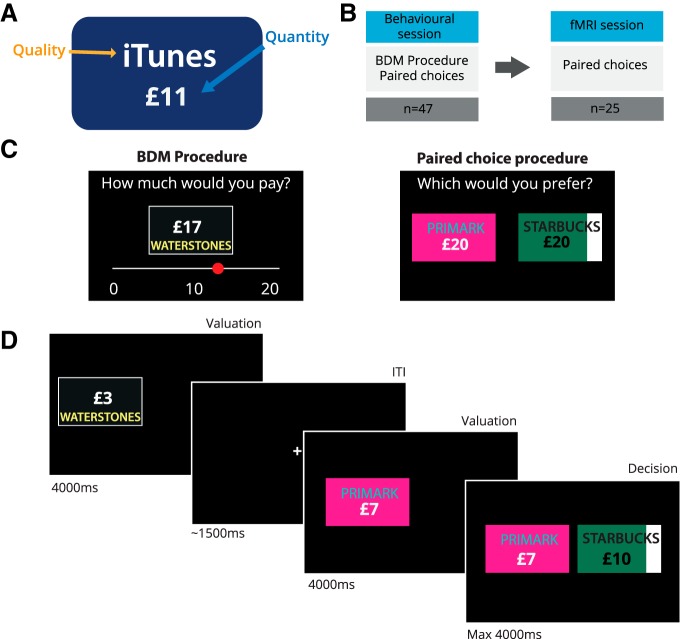
Experimental procedure. ***A***, We used giftcards to manipulate quality and quantity. Cards from different shops had different qualities, depending upon the subjective value of money that can be spent at that shop alone. Quantity varied as the amount of money (number of £) depicted on the card. ***B***, Following an initial behavioral session in which we mapped value functions for different giftcards, a subset of participants was invited to return for an fMRI session. ***C***, Behavioral experiment. The first task involved an auction (BDM procedure). Participants were offered different cards with varying amounts of money on them and indicated the maximum amount they would be willing to pay for that card. In the second (paired-choice) task, subjects made choices between pairs of giftcards with equal quantities (£20). ***D***, fMRI experiment. On most trials (six of seven), participants saw only a single giftcard from one of three different shops, with a randomly varying quantity (amount of money). On decision trials (one of seven), a second giftcard was displayed 2 s after the first, and participants had 4 s to make a choice between the two giftcards. ITIs were normally distributed at ∼1.5 s.

During the behavioral session, participants completed the following two tasks: an auction procedure, from which they could obtain a mixture of up to £20 cash and a £20 giftcard, and a session of paired choices between cards worth £20 (Fig. 1). One trial was randomly selected across both sessions and reimbursed appropriately. For the fMRI experiment, participants first completed paired choices between cards worth £20 outside of the scanner, and subsequently chose between cards worth £1–20 within the scanner. One trial from each task was reimbursed, in addition to a £20 flat rate for experiment completion.

### Experimental design and statistical analyses

#### Behavioral session

Participants first performed an auction task (BDM) designed to elicit the subjective valuation of different giftcards holding varying amounts of money (Becker et al., 1964). Briefly, the BDM involves players placing a minimum bid for an item on each trial. After the experiment, a single trial is randomly selected for reimbursement. For that trial, a randomly drawn number—the “cost”—is compared with the bid. If the cost is higher than the bid, the player retains their endowment and does not receive the item. If the bid is higher than the cost, then the player receives the item, but, crucially, pays the cost rather than their bid. This removes an incentive to place low bids, resulting in an optimal strategy whereby players report their true values. Each of 13 giftcards were presented in association with 12 different quantities, giving a total of 156 trials. Following the auction task, participants chose between pairs of giftcards of matched quantity (£20). Each combination of cards was presented twice, yielding 325 trials after the removal of trials involving two copies of the same card.

We selected a subset of participants to complete the scanning part of the study. Selection was based upon reliability, stability, and diversity of preferences over giftcards. We fit linear regressions to values reported during the auction procedure, yielding the following equation for each giftcard:

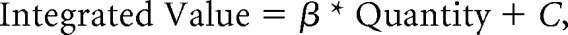
 where β and *C* (an intercept term) were fit using robust regression. The β values thus obtained are a measure of the quality of a giftcard, the value of a single unit of currency on that giftcard. We next assessed how well these β values predicted paired choice (see Fig. 3), selecting subjects for whom there was a close relationship.

For the scanning session, we selected three giftcards, which were chosen to maximize variance in quality (see Fig. 3*C*). We thus selected the lowest and highest quality card (max β and min β), and one closest to the mean of the two. Having performed this selection procedure, we verified that choices of these cards in the paired-choice session reflected the rankings calculated from the BDM (see Fig. 3). These β values were used as indicators of quality for the fMRI analyses in which parametric modulators were used [general linear model (GLM) 2 and GLM3; see below].

#### fMRI task

The task design allowed us to examine representations of quality, quantity, and their interaction using both linear analyses and measures of repetition suppression. To avoid measurements being confounded by variables related to the dynamics of stimulus comparison (Hunt et al., 2015), on the majority of trials we presented a single stimulus (Fig. 1*D*) and asked participants to evaluate its desirability. The presentation side was flipped every 10 trials. Stimuli remained onscreen for 4000 ms before being followed by an intertrial interval (ITI; normally distributed at 1500 ms) or, in one of seven trials, the appearance of a second giftcard. Participants were asked to make a choice between the two within 4000 ms, using a button box. Failure to register a choice within this time period resulted in a “TIME OUT” message, and participants were informed before scanning that if a timed-out trial was selected for reimbursement, they would receive no payment for that part of the experiment. Each giftcard displayed in the scanner was pseudocolored red or blue to reduce gross visual differences between cards.

Repetition suppression in fMRI effects can show sensitivity to expectation (Summerfield et al., 2008), necessitating counterbalancing of stimulus order. We designed our trial presentation order such that there was no relationship between the current trial and the next one. This served the dual purposes of avoiding potential confounds in our repetition suppression analysis, and ensuring consistent engagement of our subjects, who were unable to predict when they might have to make a decision.

We defined seven trial types (red and blue versions of each three giftcards + decision trials) and used a genetic algorithm to find a stimulus (stim) order in which *p*(stim*^i^*_2_|stim*^j^*_1_) was matched for all stimuli *i* and *j*. We manually removed trials on which decisions were repeated, leaving a sequence of 97 stimuli. The quantity (1–20) on the giftcards was randomized, effectively orthogonalizing quality and quantity (mean correlation coefficient across participants = 0.0074, *p* = 0.40). Participants completed four runs of the task, yielding a total of 340 stimulus evaluation trials and 48 decision trials.

#### Logistic regression modeling of choices during fMRI task

We used logistic regression to characterize the factors modulating choices in the scanner. For each participant, we fit a model to predict whether they chose the new card (presented during the decision trial) or the old card (on-screen from the valuation trial):

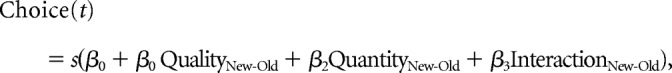
 where β_0_ is a constant term accounting for option-independent biases in choice, β_1–3_ are regression coefficients describing the effect of each term on choice, and *s* is the sigmoid function:

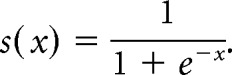
 Quality was defined using the β values from the BDM auction (see above; see Fig. 3*A*), while quantity merely reflected the monetary amount (in pounds) depicted on each giftcard. We formulate the interaction term by first normalizing quality and quantity, and then taking the product.

To assess choice predictability, we took the output of the model (valued between 0 and 1), rounded it (such that choices were either a 0 or a 1), and compared it to the vector of actual choices made by the participant. Predictability was then defined simply as the percentage of choices correctly predicted by the model.

#### fMRI data acquisition

Data were acquired using a Siemens 3 T Trio Scanner with a 32-channel head coil at the Wellcome Trust Centre for Neuroimaging. We used a 2D echoplanar image (EPI) sequence optimized to minimize dropout in the orbitofrontal cortex (OFC; Weiskopf et al., 2006), with voxels 3 mm isotropic (TR = 3.36 s, TE = 30 ms), with 48 slices giving whole-brain coverage. Slices were tilted at −30°. Scans were preceded by a field map (TE1 = 10 ms, TE2 = 12.46 ms). The first five volumes of each run were discarded to allow for T1 equilibration. We also acquired a T1-weighted structural scan for each subject, comprising 176 slices over a field of view of 256 mm with a 1 mm isotropic resolution (TR = 7.92 ms, TE = 2.48 ms; Deichmann et al., 2004). Throughout scanning, we monitored the breathing rate, using a pneumatic belt and pulse, and blood oxygenation, using an infrared pulse oximeter (Model 8600 F0, NONIN). Both were digitized and recorded via Spike2 (version 6.17), and subsequently included in GLM analyses of brain activity along with regressors derived from motion correction (Hutton et al., 2011).

#### fMRI data preprocessing

All preprocessing and data analysis took place in SPM12 (http://www.fil.ion.ucl.ac.uk/spm/). Subsequent data visualization took place in MRIcron (http://people.cas.sc.edu/rorden/mricron/index.html) and MRIcroGL (http://www.cabiatl.com/mricrogl/). Having discarding the first 5 volumes, we corrected EPIs for field inhomogeneities using acquired field maps. We then bias corrected, slice-time corrected (to the middle slice), and realigned and unwarped to the first EPI for each participant. EPIs were then co-registered to each participant's structural scan. EPIs were then coregistered to each participant's structural scan. We used the DARTEL toolbox for between-subject registration and normalization (Ashburner, 2007). Structural images were first segmented into white matter, gray matter, and CSF components. Segmented images were then iteratively warped into normalized MNI space, providing a template that was then used to normalize EPIs, a step that included Gaussian smoothing at 8 mm FWHM.

#### fMRI data analysis

Data were analyzed using a series of GLMs. These were estimated for each participant, including the calculation of contrasts between different regressors (first-level analysis). This provided summary statistics (β values) that could be tested at a population level versus a null hypothesis that they were on average equal to zero (second-level analysis; Friston et al., 1999). To obviate multiple comparisons when performing whole-brain analyses, we applied a correction using a cluster-defining threshold of *p* < 0.005, and a cluster-corrected familywise error (FWE) threshold of *p* < 0.05, except in the analysis of repetition suppression (GLM2, below), where a more lenient cluster-forming threshold of *p* < 0.01 was used, which is in line with recent repetition suppression studies (Barron et al., 2013; Garvert et al., 2015; Boorman et al., 2016). To extract the parameter estimates displayed in Figure 5, we used group-functional ROIs thresholded at *p* < 0.005. For the conjunction analysis described in Figure 5, we took the product of three binary masks (quality, quantity, and interaction), each thresholded at *p*_uncorrected_ < 0.05, resulting in an FWE rate of *p*_uncorrected_ = 0.000125.

##### GLM1: quality and quantity.

Our first GLM incorporated separate onset regressors for cards of different qualities (low, medium, and high). Each of these was modeled as a 4-s-long boxcar and was associated with a parametric modulator corresponding to the quantity on the card at each presentation. We used a fourth-onset regressor corresponding to decision trials, which were modeled as Δ functions. This GLM was used to perform a whole-brain analysis of value computations during evaluation trials.

We performed the following three key contrasts:

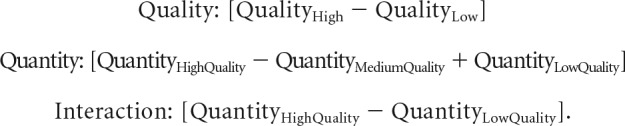
 The interaction analysis was constructed to test for regions displaying steeper coding of quantity for high-quality cards compared with low-quality cards, consistent with value integration. This corresponds to an intuition that an extra unit of a more desirable good (e.g., a Ferrari) is worth more than an extra unit of a less desirable good (e.g., an apple). We excluded trials preceding decisions from the evaluation regressors to guard against contamination of the evaluation regressors by decision-related activity, a possibility arising out of the lack of ITI between evaluation and decision trials.

##### GLM2: repetition suppression.

Following the numerosity-coding literature (Piazza et al., 2004, 2007; Jacob and Nieder, 2009), we designed a repetition–suppression analysis based upon the absolute change in value between trials (see Fig. 7*A*). We used this analysis to reveal repetition suppression effects within ROIs identified by the whole-brain analysis using GLM1.

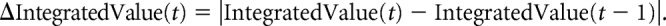
 where IntegratedValue(*t*) is simply the product of quality and quantity on trial *t*. We used a single-onset regressor to represent all giftcard presentations, again using a 4 s boxcar, with parametric modulators for ΔIntegrated Value, and, as a precaution, Integrated Value. The inclusion of Integrated Value in the model allowed us to confirm that the effects of ΔIntegrated Value were not simply the result of spurious correlation with Integrated Value itself. Trials following decisions were excluded as they were preceded by a pair of stimuli, obfuscating the calculation of stimulus similarity. As before, we used a second-onset regressor for decision trials. Contrasts were calculated merely as the value of the relevant parametric modulators.

##### GLM3: integrated value.

To obtain a measure of integrated value coding, we used a single 4 s boxcar for all evaluation trials, which was associated with a parametric modulator for integrated value (Quality × Quantity), excluding predecision trials as in GLM1. As in GLM1 and 2, decision trials were modeled in a separate regressor with Δ onsets. We used this analysis within ROIs identified by the whole-brain analysis in GLM1, to confirm that the ACC region showing a conjunction of quality, quantity, and interaction effects could also be described as coding-integrated value.

##### Statistical tests.

Parameter estimates from fMRI are normally distributed, permitting the use of parametric statistics (*t* tests and Pearson correlations). When analyzing distributions that we knew a priori to be non-normal (e.g., predictability, which is bounded at 0 and 100), we used nonparametric equivalents (sign tests and Spearman rank coefficients). All statistical testing was performed in Matlab.

## Results

### Behavioral session establishes stable quality estimates

We used a behavioral session to identify participants for whom we could find giftcards with consistently different subjective qualities (Fig. 1*B*). The behavioral session consisted two tasks. Participants (*n* = 47) performed a BDM auction (Becker et al., 1964) and a series of paired choices, each involving a selection of 13 giftcards (Fig. 1*C*). In the BDM auction, players reported how much they would be willing to pay for a giftcard loaded with a certain amount of money, from £1 to £20. Subsequently, participants made paired choices between different giftcards containing matched sums (£20).

**Figure 2.**
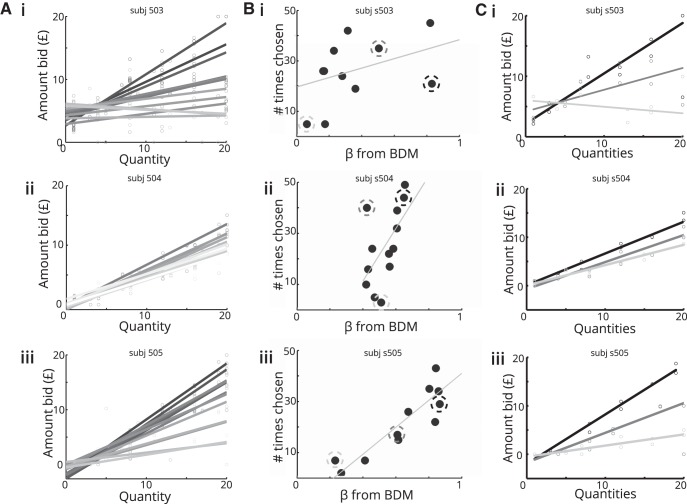
Example participants from behavioral experiment. We used data from the behavioral session to determine subsequent inclusion in an fMRI study based on criteria of consistency and diversity of preferences for different giftcards (assessed using the predictability of BDM ratings), relationships between BDM and paired-choice tasks, and correlations between quantity and subjective value. ***A***, First, we examined quantity–bid relationships for the 13 different giftcards. The slope of the quantity–bid relationship for each giftcard is a measure of that the quality of the giftcard, with higher slopes corresponding to more valuable brands. Here, the participant in ***i*** has diverse but noisy preferences, the participant in ***ii*** is consistent but has similar preferences across giftcards, while the participant in ***iii*** displays an acceptable level of consistency while maintaining diverse preferences. ***B***, To assess preference stability, we compared the slope of lines estimated from the BDM task with the number of times each giftcard was chosen in the paired-choice task. The participant in ***i*** shows a weak relationship between choices in each session; the participant in ***ii*** is consistent but shows little variability; and the participant in ***iii*** is both consistent and displays diverse preferences. ***C***, For the fMRI experiment, we selected three giftcards for each participant that differed maximally in quality. Here we show BDM plots for selected cards. As before, ***i*** is noisy but shows diverse quality preferences, ***ii*** has similar preferences over giftcards, and ***iii*** has consistent and diverse preferences over giftcards.

We used a linear fit to the relationship between the amount of money on the giftcard and the amount bid for each giftcard during the BDM to provide a measure of the quality of each giftcard for each participant (Fig. 2*A*). To maximize power in the fMRI study, we selected subjects whose bids were predictable (Fig. 2*A*) and for whom we could select three giftcards with distinct qualities (Fig. 2*B*, circled points, *C*). By way of confirmation that BDM-estimated values predicted choice, we next compared quality estimates from the BDM with the number of choices of each giftcard in the paired-choice session, preferring subjects for whom there was a high correlation (Fig. 2*B*).

**Figure 3.**
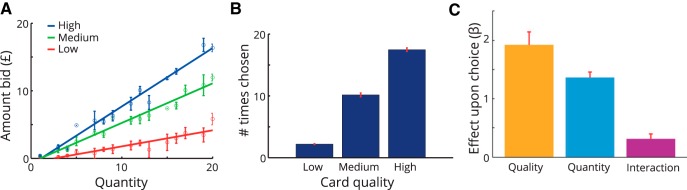
Behavioral results for subjects in scanning experiment. ***A***, Average quantity–bid functions show the difference in quality for three selected giftcards for subjects who completed both the behavioral and fMRI sessions (*n* = 25). ***B***, In a prescanning paired-choice session, we confirmed that the ordering of cards by quality was highly consistent between sessions. ***C***, Analysis of choices made in the MRI scanner. During the fMRI experiment, participants made 48 choices between cards of varying quality and quantity (Fig. 1*D*). We used the differences between options to predict choices using logistic regression. The differences between options in both quality (*t*_(24)_ = 8.6, *p* < 0.001) and quantity (*t*_(24)_ = 13.7, *p* < 0.001) were predictive of choice. Importantly, the interaction between quality and quantity also predicted choice (*t*_(24)_ = 3.8, *p* < 0.001), consistent with the multiplicative relationship expected from the observed quantity–utility functions (***A***). Errors bars represent the SEM across subjects.

Selected subjects thus displayed consistent BDM bids, a high correlation between preferences elicited in the BDM and paired-choice sessions, and a low maximum correlation among quality, quantity, and integrated value (Fig. 3). Integrated value, calculated as the product of quality and quantity, effectively provided a prediction of the bid a participant would place for a given giftcard. The correlation among quality, quantity, and integrated value reflects the diversity of giftcard qualities. Giftcards with disparate qualities limit the correlation between quantity and integrated value (Fig. 2*C*, *i*, *iii*), while if all giftcards have similar qualities, the quantity/integrated value correlation will be high (Fig. 2*C*, *ii*).

### fMRI experiment: subjects integrate quality and quantity in choice

For each participant in the fMRI experiment (*n* = 25), we used data from the behavioral session to select the following three giftcards: the giftcard that displayed the steepest relationship between BDM bid and quantity (high quality); the giftcard that displayed the lowest (low quality); and a giftcard of intermediate slope (medium quality; Fig. 3*A*). In a prescanning paired-choice session, we confirmed that preference estimates from the preceding behavioral session were stable, with subjects making choices among the three selected giftcards in a highly predictable manner (Fig. 3*B*).

Within the scanner, participants made choices between giftcards of varying quality and quantity on one of seven trials (Fig. 1*D*), resulting in a total of 48 decisions. Participants remained highly engaged throughout, exceeding the time limit for choice of 4000 ms in only 7 of 1200 choices. We used a logistic regression analysis to quantify the impact of differences between the two options upon choice. We calculated an interaction term as the mean-centered product of quality and quantity. Intuitively, the interaction term captures the fact that an extra pound on the highest quality giftcard is more valuable to the subject than an extra pound on the low-quality giftcard. Differences among options in quality, quantity, and their interaction all influenced participants' choices (quality: *t*_(24)_ = 8.6, *p* < 0.001; quantity *t*_(24)_ = 13.7, *p* < 0.001; interaction: *t*_(24)_ = 3.8, *p* < 0.001; Fig. 3*C*), implying that participants combined information about quality and quantity to estimate integrated subjective value, rather than considering the two attributes independently.

### Brain activity associated with quality, quantity, and their interaction

In the scanner, participants were shown a single giftcard and asked to internally evaluate it (evaluation trials), in the knowledge that they might have to make a fast decision between that option and another (decision trials; Fig. 1*D*). The preponderance of valuation trials (340 of 388) provided us with an opportunity to examine value computation in isolation, without potentially confounding effects of decision dynamics (Hunt et al., 2015).

To isolate elements of value representation, we used GLMs of voxelwise brain activity to examine the representation of quality, quantity, and their interaction in valuation trials. We split card presentations by quality (low, medium, high) and associated each onset with a parametric modulator corresponding to the quantity presented on that trial. This allowed us to index the main effects of card quality (Quality_High_ − Quality_Low_, card quantity (Quantity_LowQuality_ + Quantity_MediumQuality_ + Quantity_HighQuality_), and the interaction between the two (Quantity_HighQuality_ − Quantity_LowQuality_).

The interaction term allows us to identify regions where quantity affects activity more when giftcard value is high compared with when it is low. By decomposing value in this way—into quality, quantity, and their interaction—we can identify brain regions displaying specific relationships with each component, as well as regions showing an overlap of all three effects. This conjunction analysis is more stringent than a simple contrast for integrated value, because it prevents erroneously identifying regions that simply have a strong correlation with only quality or quantity. Motivated by this same logic, recent studies formulate fMRI contrasts for reward prediction errors (RPEs) as a conjunction of positive coding for reward and negative coding for reward expectation, thus avoiding false-positive results arising from the correlation between RPEs and other variables such as reward itself (Rutledge et al., 2010).

**Figure 4.**
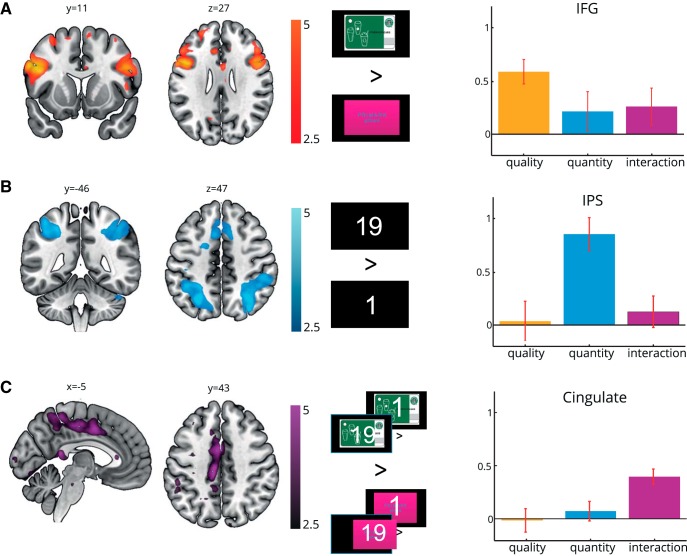
Representation of quality, quantity, and their interaction. ***A***, We observed bilateral coding of offer quality (Quality_High_ − Quality_Low_) bilaterally in the IFG. ***B***, Increasing quantity, as tested by Quantity_HighQuality_ + Quantity_MediumQuality_ + Quantity_LowQuality_, was associated with greater activity bilaterally in the IPS. ***C***, Activations in the posterior cingulate cortex were consistent with representing the interaction of quality and quantity but not either variable separately (Quantity_HighQuality_ − Quantity_LowQuality_). Errors bars represent the SEM across subjects, SPM values were thresholded at *p* < 0.01 for visualization.

We found three largely nonoverlapping patterns of response corresponding to the representation of offer quality, quantity, and their interaction. Higher card quality was associated with greater activity in bilateral IFG, centered on the pars opercularis (left: peak MNI = −54, 12, 30; *t*_(24)_ = 4.79, *p*_FWE-corrected_ = 0.023; right: peak MNI = 51, 9, 27; *t*_(24)_ = 4.37, *p*_FWE-corrected_ = 0.032; Fig. 4*A*). On the left, this extended into dorsolateral PFC (peak MNI = −36, 48, 24; *t*_(24)_ = 3.16, *p*_FWE-corrected_ = 0.044) and included Broca's area, an area associated with semantic comprehension (Price, 2012), arguably a process necessary for evaluating abstract stimuli such as giftcards. Parameter estimates extracted from group-level functional ROIs within IFG (defined at *p* < 0.005) suggested an absence of sensitivity to either quantity (*t*_(24)_ = 1.18, *p* = 0.24) or the interaction of quantity and quality (*t*_(24)_ = 1.53, *p* = 0.14) in this region, although direct comparisons did not distinguish quality coding from that of quantity or the interaction (quantity: *t*_(24)_ = 1.88,*p* = 0.073; interaction: *t*_(24)_ = 1.88,*p* = 0.17).

Offer quantity correlated with activity in bilateral IPS (Left: peak MNI, −27, −66, 51; *t*_(24)_ = 4.77, *p*_FWE-corrected_ < 0.001; right: peak MNI, 33, −66, 51; *t*_(24)_ = 4.68, *p*_FWE-corrected_ < 0.001) resonating with a role for this region in numerical reasoning in humans and nonhuman primates (Nieder and Miller, 2004; Piazza et al., 2004, 2007; Pinel et al., 2004; Harvey et al., 2013; Fig. 4*B*). As for IFG, activity in the IPS was selective for number, with no sensitivity to quality (*t*_(24)_ = 0.19, *p* = 0.85) or the interaction of quality and quantity (*t*_(24)_ = 0.84, *p* = 0.41). This indicates that IPS is not performing value coding per se, but specifically represents the quantity of available options. Direct comparisons confirmed that quantity correlations were greater than those for quality (*t*_(24)_ = 3.52, *p* = 0.0017) and for the interaction (*t*_(24)_ = 3.36, *p* = 0.0025). We also observed quantity-related activity in bilateral visual cortex (left: peak MNI, −33, −87, −12; *t*_(24)_ = 5.54, *p*_FWE-corrected_ < 0.001; right: peak MNI, 27, −87, −12; *t*_(24)_ = 5.49, *p*_FWE-corrected_ < 0.001).

Finally, we asked whether activity in any region of the brain was associated with an interaction between quality and quantity, correlating more steeply with quantity for high-quality giftcards compared with low-quality giftcards. This is a signature of value computation, involving additional processing above and beyond a simple reflection of option quality or quantity. The most prominent effect was located along the posterior cingulate cortex (peak MNI, −12, −15, 54; *t*_(24)_ = 3.57, *p*_FWE-corrected_ < 0.001) where activity was specific to the interaction term, with no evidence of quality (*t*_(24)_ = −0.14, *p* = 0.89) or quantity (*t*_(24)_ = 0.77, *p* = 0.45) correlations, implying that despite the involvement of this region in value computation, it does not represent an integrated value signal per se. Direct comparisons confirmed that the interaction effect exceeded both quality (*t*_(24)_ = 2.93, *p* = 0.0072) and quantity (*t*_(24)_ = 2.53, *p* = 0.018) contrasts. Interaction contrast effects were also present in bilateral superior temporal lobes (left: peak MNI, −63, −45, 0; *t*_(24)_ = 5.55, *p*_FWE-corrected_ < 0.001; right: peak MNI, 48, −33, 3; *t*_(24)_ = 4.85, *p*_FWE-corrected_ < 0.001; Fig. 4*C*).

### Computation of integrated value from component parts in the cingulate

Having characterized neural responses to individual components of option value (quality, quantity, and their interaction), we next asked whether any regions represented integrated value. To do so, we formulated a parametric regressor for subjective integrated value, by combining quality and quantity mutliplicatively in the manner suggested by our behavioral results (Fig. 3).

However, since integrated value is correlated with quality and quantity (though this correlation is limited by design) testing for effects of integrated value presents a problem as regions sensitive to quality or quantity alone might appear to reflect integrated value. To overcome this, we supplemented our parametric analysis with a conjunction analysis, reasoning that a region truly representing integrated value ought to display sensitivity to all of its components: quality, quantity, and their interaction. Importantly, the interaction of two mean-centered variables is decorrelated from either component, giving us a way to check for correlates of the computation of subjective value.

**Figure 5.**
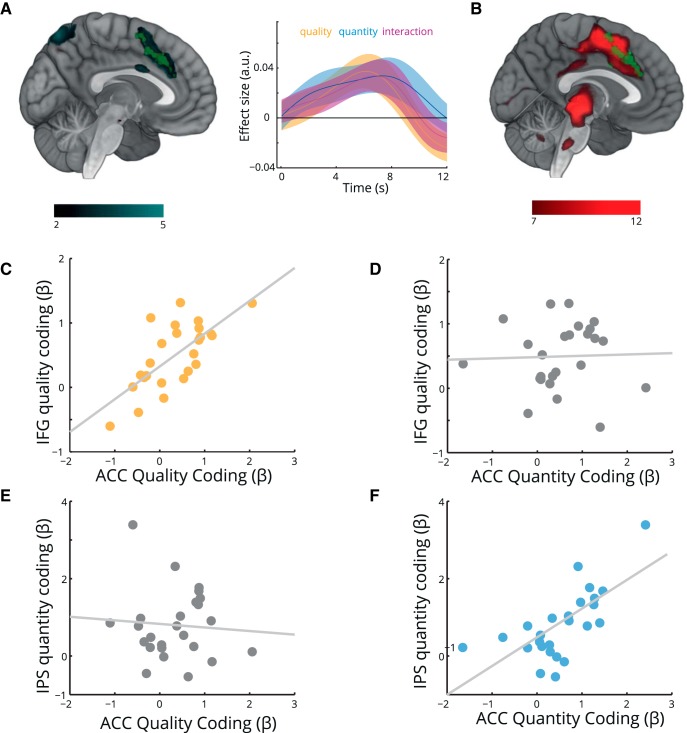
Computation of value from component parts in the anterior cingulate cortex. ***A***, Overlapping effects of quality, quantity, and their interaction in the ACC. A conjunction analysis revealed overlapping representations of each component (*p*_uncorrected_ < 0.05; green) in the ACC, suggesting a nexus for the computation of value. A complementary analysis using an explicit representation of integrated value as a parametric modulator identified the same region (*p*_FWE-corrected_ < 0.001; turquoise). Time course displayed for illustration purposes. ***B***, Decision > nondecision trials. The ACC also showed higher activity in trials upon which a decision was made compared with valuation trials (red), overlapping with the conjunction analysis identified in ***A*** (green). ***C***, Participants with stronger representations of quality in the IFG showed stronger representations of quality in the ACC (*r* = 0.63, *p* < 0.001). ***D***, Quality sensitivity in IFG was unrelated to quantity coding in ACC (*r* = 0.04, *p* = 0.83). ***E***, Quantity sensitivity in IPS was unrelated to quality coding in ACC (*r* = −0.14, *p* = 0.50). ***F***, Participants with stronger representations of quantity in the IPS showed stronger representations of quantity in the ACC (*r* = 0.68, *p* < 0.001). Each point is one participant.

Both analyses revealed a striking convergence on the ACC (Fig. 5) where activity covaried with a parametric modulator for integrated value (peak MNI, −12, 21, 39; *t*_(24)_ = 6.04, *p*_FWE-corrected_ < 0.001) and showed a conjunction of effects of quality, quantity, and their interaction (all *p* < 0.05_uncorrected_). This chimes with known roles of this regions, including the fact that it contains neurons that multiplex attributes in value-based decision-making (Kennerley et al., 2011) and its necessity for value learning (Rushworth and Behrens, 2008; Hayden et al., 2009). Decomposing the interaction effect within the ACC, we observed that although quantity coding for all three qualities was positive, it was only significantly so in the high-quality condition (Quantity_LowQuality_: *t*_(24)_ = 0.15, *p* = 0.87; Quantity_MidQuality_: *t*_(24)_ = 1.51, *p* = 0.14; Quantity_HighQuality_: *t*_(24)_ = 4.24, *p* < 0.001). By way of comparison, all three of these effects were significant in the IPS region pictured in Figure 4*B*, with no significant difference between coding of quantity for high-quality and low-quality giftcards (Quantity_LowQuality_: *t*_(24)_ = 2.30, *p* = 0.03; Quantity_MidQuality_: *t*_(24)_ = 2.69, *p* = 0.012; Quantity_HighQuality_: *t*_(24)_ = 4.78, *p* < 0.001; Quantity_HighQuality_ vs Quantity_LowQuality_: *t*_(24)_ = 1.17, *p* = 0.25).

We next reasoned that if the ACC value estimates guide choice, then we should see greater activity in decision trials compared with valuation trials. This was indeed the case with decision trials associated with enhanced activity in the same region (peak MNI, 9, 15, 45; *t*_(24)_ = 17.03, *p*_FWE-corrected_ < 0.001; Fig. 5*B*). Dorsally, this region overlaps with activity in dorsomedial PFC (dmPFC) showing an integrated value difference signal (Lebreton et al., 2009; Grueschow et al., 2015) and previously characterized as the final value comparison step before motor output (Hare et al., 2011).

Our analyses revealed dissociable representations of quality, in the IFG, and quantity, in the IPS. Although we lack the temporal precision to test whether these segregated representations precede the emergence of integrated value signals in ACC, we nevertheless can ask whether between-subject variability in component coding is related to between-subject variability in ACC representations. We found evidence that this was the case, with stronger IFG encoding of quality associated with stronger coding of quality in ACC (*r* = 0.63, *p* < 0.001; Fig. 5*C*), while stronger IPS encoding of quantity was associated with stronger quantity coding in the ACC (*r* = 0.68, *p* < 0.001; Fig. 5*F*). Importantly, the converse correlations did not hold, with parameter estimates for IGF quality unrelated to ACC quantity (*r* = 0.04, *p* = 0.83) and IPS quantity unrelated to ACC quality (*r* = 0.14, *p* = 0.50) coding (Fig. 5*D*,*E*). This specificity suggests that observed correlations reflect meaningful inter-regional relationships rather than correlated variance in signal-to-noise ratio between participants.

### Strength of neural quantity coding reflects choice predictability

**Figure 6.**
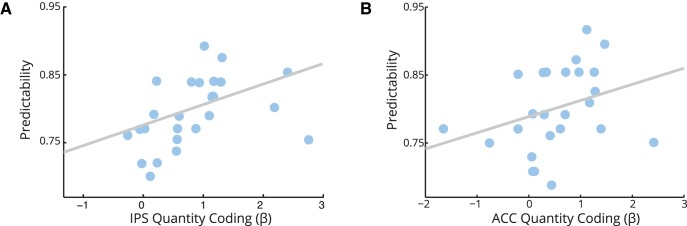
***A***, ***B***, Neural quantity sensitivity relate to choice predictability. We found that coefficients for quantity in IPS (ρ = 0.60, *p* = 0.002; ***A***) and the ACC (ρ = 0.42, *p* = 0.039; ***B***) correlated with the predictability of participants' choices, as assessed by the ability of our logistic regression model to predict choice. Correlations with quality coding in the IFG (ρ = 0.41, *p* = 0.104) and ACC (ρ = 0.30, *p* = 0.142) were positive but not significant. Each point is one participant.

The degree to which subjects' choices were correctly predicted by our logistic regression varied from 69% to 92%. We reasoned that stronger neural representations of value components should lead to more predictable choices. Using parameter estimates (β values) extracted from our GLM analyses, we asked whether between-subject variability in β values related to between-subject choice predictability. We found that the strength of neural correlations with quantity, but not quality, was associated with predictability of choice (Fig. 6). Mean β values in both the IPS (ρ = 0.60, *p* = 0.002) and ACC (ρ = 0.42, *p* = 0.039) were positively correlated with choice predictability, suggesting that stronger neural representations of quantity correspond to more reliable choices. Correlations between predictability and quality coding in the IFG (ρ = 0.41, *p* = 0.104) and ACC (ρ = 0.30, *p* = 0.142) were also positive but did not reach significance, perhaps reflecting the greater range of values for quantity than quality or the potential impact of overtraining on giftcard quality. We observed no relationship between interaction coding in ACC and predictability (ρ = −0.04, *p* = 0.83). We note that after applying a conservative Bonferroni correction for the five comparisons we make here, only the effect in the IPS survives an adjusted threshold of α = 0.01.

Using the summed log-likelihood of choices according to the logistic regression model as an alternative measure of choice predictability yielded a consistent pattern of results, although the effect in the ACC was no longer significant at α = 0.05 (IPS: ρ = 0.55, *p* = 0.004; ACC: ρ = 0.35, *p* = 0.089). We did not observe any other correlations between the parameters of our logistic regression model for behavior and those of our GLMs for neural activity.

### Repetition suppression for integrated value in the ACC

**Figure 7.**
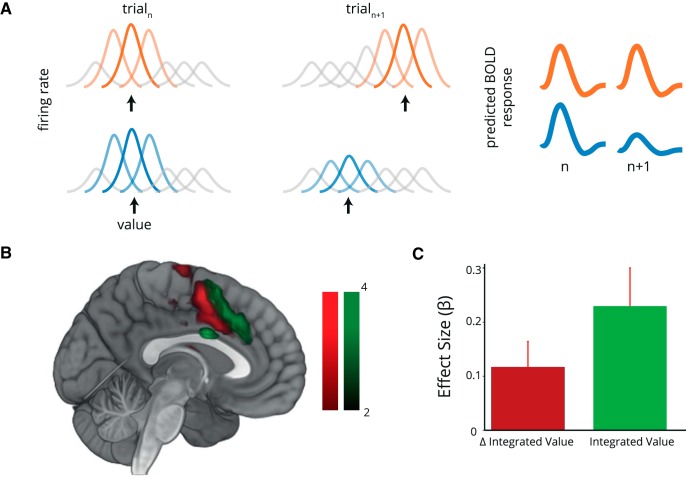
Repetition suppression for value in the anterior cingulate cortex. ***A***, Repetition suppression analysis logic. We hypothesize a population of neurons tuned to value where the different neurons have overlapping tuning curves spanning the range of values presented. Black arrows denote stimulus value for that trial. If consecutive trials activate nonoverlapping populations of neurons, evoked responses for each stimulus are similarly high on each trial (top panel in orange). However, repeated presentation of the same stimulus produces repeated activation of the same neurons on consecutive trials, leading to a reduction in the neural response (bottom panel in blue). Summation over all neurons in the population (as in the BOLD signal measured in fMRI), leads to higher activity when consecutive stimuli activate unique subsets of neurons (top panel) than when consecutively activated populations overlap (bottom panel). Predicted BOLD activity is thus proportional to the absolute difference in value between consecutive trials. ***B***, Evidence for multiple forms of value coding in the cingulate. We examined cingulate representations of repetition suppression to integrated value (change in value from trial *n* − 1 to trial *n*, ΔIntegratedValue; green), and monotonic encoding of integrated value (a standard parametric modulator approach; red). Voxels sensitive to repetition suppression were more posterior, with monotonic encoding stronger in anterior voxels. ***C***, The ACC region identified in the conjunction analysis (Fig. 5*A*) also shows repetition suppression in the integrated value. We extracted mean parameter estimates for ΔIntegratedValue and for integrated value from the voxels identified in the conjunction analysis. Both were positive on average (ΔIntegratedValue: *t*_(24)_ = 2.48, *p* = 0.020; Integrated Value: *t* = 3.26, *p* = 0.0034). Error bars represent the SEM across participants.

RS describes the phenomenon whereby repeated presentation of stimuli that are similar along some dimension evoke reduced activity in brain regions sensitive to that attribute (Grill-Spector et al., 2006). This is putatively due to a reduction in activity in neurons activated in both trials (Fig. 7). This provides a means to assay the neural overlap in the representation of two stimuli such as foods (Barron et al., 2013), faces (Loffler et al., 2005), or even agents (Garvert et al., 2015). This can reveal nonmonotonic codes invisible to traditional GLM approaches, such as tuned numerosity representations in the parietal cortex (Piazza et al., 2004, 2007; Jacob and Nieder, 2009). Since our task involved the calculation of value from information about quantity, we hypothesized that value representations in the ACC might show a similar form.

We asked whether RS provides additional evidence of value encoding in ACC. We constructed a GLM where we modeled the absolute difference in integrated value (ΔIntegratedValue) between subsequent trials, as well as the Integrated Value on each trial. If neurons in a brain region are undergoing RS, aggregate activity as assayed by BOLD should covary with the absolute difference between trials (Barron et al., 2016). We found evidence for repetition suppression to value in dorsal ACC (peak MNI, −3, 3, 51; *t*_(24)_ = 3.86, *p*_FWE-corrected_ = 0.003), just posterior to the activity related to monotonic encoding of integrated value (Fig. 7*B*). These activations were partially overlapping, such that the integrated value coding conjunction identified in Figure 5 showed effects of both ΔIntegratedValue and Integrated Value (Fig. 7*C*; ΔIntegratedValue: *t*_(24)_ = 2.48, *p* = 0.020; Integrated Value: *t* = 3.26, *p* = 0.0034). Repetition suppression for integrated value was surprisingly widespread. We also observed repetition suppression for value bilaterally in the lingual gyrus (left: peak MNI, −15, −45, −9; *t*_(24)_ = 6.18, *p*_FWE-corrected_ < 0.001; right: peak MNI, 15, −48, 3; *t*_(24)_ = 4.56, *p*_FWE-corrected_ < 0.001), the right superior temporal sulcus (peak MNI, 63, −30, 3; *t*_(24)_ = 5.80, *p*_FWE-corrected_ < 0.001), and bilaterally in the posterior insula (left: peak MNI, −30, 0, −3; *t*_(24)_ = 5.00, *p*_FWE-corrected_ < 0.001; right: peak MNI, 33, −6, −12; *t*_(24)_ = 3.60, *p*_FWE-corrected_ < 0.001).

## Discussion

Value representations are often studied as monolithic entities. Indeed, considerable effort has been expended in identifying abstract behavioral and neural signatures of scalar value estimates. However, recent work suggests that during choice components of value compete at an attribute level to guide decisions (Hunt et al., 2014), emphasizing the importance of decomposing value into its constituent parts. Here we show that, in the absence of choice, integrated value correlates appear in the ACC, with component representations in the IFG (quality) and IPS (quantity; Figs. 5, 6). A distinct network appears to integrate the two, with posterior cingulate and superior temporal lobe activations corresponding to the interaction between quality and quantity (Fig. 4). A more posterior region of the ACC displays repetition suppression to integrated value (Fig. 7).

### Correlates of quality and quantity in the brain

Bilateral IFG activity scaled with the quality of the giftcard presented on each trial (Fig. 4*A*). This was unexpected, given the scarcity of reports of IFG involvement in value-based decision-making (but see Rogers et al., 1999; Zysset et al., 2006; Liljeholm et al., 2011). A priori, the OFC might represent a more promising candidate for the representation of stimulus quality. However, representations in the OFC appear to be particularly entangled with stimulus identity (Padoa-Schioppa and Assad, 2008; Noonan et al., 2011; Barron et al., 2013; Klein-Flügge et al., 2013; McNamee et al., 2013; Howard et al., 2015), potentially reflecting the central role of the OFC in providing an internal model of the world (Wilson et al., 2014). For instance, Padoa-Schioppa and Assad (2008) describe OFC cells that respond specifically to one juice or another, which they describe as reflecting the taste of a given juice. This encoding of juice identity is distinct from the reward quality, and no study has reported OFC unit responses that reflect quality alone (i.e., the preference ordering of different stimuli), while being insensitive to quantity. It seems, therefore, that the OFC is particularly interested in tracking relationships between specific rewards and their predictors (Noonan et al., 2011; Takahashi et al., 2013; Stalnaker et al., 2014, 2015; Lopatina et al., 2015; Lucantonio et al., 2015; Boorman et al., 2016), rather than estimating stimulus quality per se. Furthermore, a recent study (Schuck et al., 2016) found that OFC exclusively represented hidden variables related to the current state. The lack of OFC involvement in our task is likely to reflect the static and transparent relationship between stimuli and outcomes in our experiment.

The involvement of the IFG in the representation of stimulus quality is consistent with the semantic nature of the giftcard stimuli we used. IFG is commonly activated in lexical tasks (Price, 2012), with left hemisphere lesions to this area producing impairments in language production and comprehension. In one of the few studies attempting to parse value into distinct components, Lim et al. (2013) offered participants T-shirts that varied in their esthetic and semantic properties. They found correlations with esthetic value in the fusiform gyrus and semantic value in the superior temporal gyrus, while ventromedial PFC (vmPFC) activity correlated with the value of both attributes. This suggests that the extraction of quality may occur in concert across brain areas specialized for the analysis of distinct stimulus features, in the same way that feedforward models of visual inputs eventually produce value estimates in deep reinforcement learning networks (Mnih et al., 2015; Silver et al., 2016). This suggests that a representation of stimulus quality in IFG may be specific to semantically rich stimuli, such as those used here.

Conversely, our observation of quantity coding in the IPS (Fig. 4*B*) is predicted from the literature (Nieder, 2016). A wide variety of animals show an ability to make ethologically relevant decisions using number, from lions (McComb et al., 1994) to crows (Rahman et al., 2014). Even newborn chicks are capable of tracking the number of an imprinted object that is placed behind a screen (Rugani et al., 2009). In macaques, such judgments rely upon a network of frontal and parietal regions containing neurons tuned to different numbers, including the number zero (Nieder et al., 2002; Nieder and Miller, 2004; Ramirez-Cardenas et al., 2016).

Studies in humans have made use of model-based decoding analyses (Harvey et al., 2013) and repetition suppression designs (Piazza et al., 2004, 2007; Jacob and Nieder, 2009) to provide evidence that similar tuning curves for number exist in the human IPS. Our results imply that the same IPS circuitry subserves number representation in value computation. This is consistent with the recent observation that when number and value are decorrelated, the IPS tracks quantity and not value (Kanayet et al., 2014). This serves to clarify the role of parietal cortices in value-based decision-making, suggesting that when financial stimuli are used (Ballard and Knutson, 2009; Clithero et al., 2009; Chau et al., 2014), evaluation occurs within a financial framework (e.g., the BDM auction; Plassmann et al., 2007; Medic et al., 2014), or, if stimuli merely differ in magnitude (Louie et al., 2011), parietal responses to quantity may be misconstrued as representing value or its comparison. Conversely, we find that the IPS specifically represents the quantity of an available option, and that the strength of numerical representations in IPS correlates both with choice predictability and ACC quantity coding. This is consistent with neurons in IPS contributing to the representation of stimulus value in the ACC, and this latter representation subsequently being used to guide choice. However, it is unclear why choice predictability is so much more strongly linked to quantity coding than it is to quality coding (in IFG and ACC) and to interaction coding (in the ACC). We suspect that the greater range of quantities than qualities in our experiment may have increased our power to observe relationships with quantity coding, but this remains an open question.

### The role of the ACC in evaluation

We found that activity in the ACC was consistent with the representation of integrated value. ACC showed a positive correlation with integrated value. Even after accounting for the effects of quality and quality, ACC tracked the interaction term characteristic of integrated value in this task (Figs. 5*C*, 6*A*). Beckmann et al. (2009) parcellated the cingulate cortex according to connectivity. The region we identify corresponds to their region 4, which shows strong connectivity to dorsolateral prefrontal cortex, and is commonly implicated in value-based tasks. The region showing repetition suppression effects may be more situated in their region 5, which has a higher connectivity to the parietal cortex. This raises the possibility that the repetition suppression we observe is inherited from tuned numerical representations in parietal cortex (Nieder and Miller, 2004).

The ACC is frequently identified in both human (Bush et al., 2002; Kolling et al., 2012; Boorman et al., 2013) and animal experiments (Seo and Lee, 2007; Hayden et al., 2009; Hayden and Platt, 2010; Kennerley et al., 2011; Cai and Padoa-Schioppa, 2012) of value-based choice. The more dorsal region in which we find signatures of integrated value is associated with tasks wherein participants assign value to actions (Beckmann et al., 2009). This is the case in our experiment, since giftcards were displayed either on the left-hand or the right-hand side of the screen, such that assessing the value of a particular giftcard was the same as assessing the value of a left/right button press. Dorsal ACC appears to be particularly engaged by foraging type tasks, in which the pertinent comparisons are between options presented sequentially (Seo and Lee, 2007; Kolling et al., 2012; Boorman et al., 2013). We further note that since the positive value correlations we observe in the ACC are recorded in the absence of choice, they cannot be explained as a function of choice difficulty and are more consistent with a proposed role in sequential foraging decisions (Kolling et al., 2016).

We did not observe value-related activity in the vmPFC, the part of the cortex most frequently associated with valuation (Rushworth and Behrens, 2008), or in the ventral striatum. This agrees with recent observations suggesting that sequential (Hunt et al., 2013) or time-limited (Jocham et al., 2014) choices do not engage vmPFC. Indeed, a growing body of evidence suggests that the ACC is particularly involved when subjects make sequential, foraging-type decisions, which are characterized by an evaluation of whether to engage or not (Kolling et al., 2012, 2016). Conversely, whether evaluation alone effectively engages vmPFC is unclear. Although early reports suggested that the vmPFC was part of an automatic valuation system (Lebreton et al., 2009), recent work suggests otherwise (Grueschow et al., 2015). The few studies that report value-related activity in macaque vmPFC do so in the context of free viewing (Strait et al., 2014; Abitbol et al., 2015), raising the possibility that the vmPFC is particularly engaged when values are compared via repeated eye movements (Krajbich et al., 2010). The observation that vmPFC is crucial for episodic memory and imagination (Hassabis and Maguire, 2009; Benoit et al., 2014), and a predominance of saccade-frequency theta oscillations in mPFC (Paz et al., 2008; Adhikari et al., 2010) hints at a more general role for the vmPFC in mediating a short-term plasticity allowing features—of a scene, an episode, or a choice—to be integrated over several seconds. This might explain why our task, which required participants to evaluate a single stimulus at a single location, did not modulate vmPFC activity.

Our finding that the cingulate cortex integrates information about quality and quantity to form a multiplicative value representation of the current stimulus is also interesting in light of a literature implicating the cingulate in the representation of values associated with “model-based” cognition (Wunderlich et al., 2012; Doll et al., 2015). This describes flexible computation of value associated with a certain stimulus, and is typically contrasted with “model-free” cognition, in which stimulus or action values are cached and updated only through repeated experience (Dolan and Dayan, 2013). The multiplication of quantity and quality that we observe in the ACC is consistent with the idea that the cingulate provides a model that produces estimates of quantities relevant to behavior (O'Reilly et al., 2013; Economides et al., 2014; Kolling et al., 2014). In our case, utility was maximized by combining quality and quantity in a multiplicative manner, and this is what the ACC appears to do, in a manner that reflects the coding of quality and quantity in the frontal and parietal lobes respectively (Fig. 5*C*,*F*).

Our design also enabled us to perform a repetition suppression analysis, allowing us to reveal coding schemes hidden to conventional BOLD analyses. We found that parts of the cingulate cortex displayed repetition suppression to integrated value, with activity that scaled with the absolute difference in value between trials (Fig. 7*A*). This region was posterior to the peak activity associated with monotonic integrated value, extending into the area identified in the conjunction analysis of quality, quantity, and their interaction (Fig. 7*B*). Although the precedent from the numerosity-coding literature is to suppose that RS results of this kind provide positive evidence of nonmonotonic tuning (Piazza et al., 2004, 2007; Ansari and Dhital, 2006; Jacob and Nieder, 2009), we are cognizant that such repetition suppression effects are not an unambiguous signature of nonmonotonic codes. In modeling work reported previously, we observe that repetition suppression effects such as the ones we observe here can result from mixed linear codes combined with divisive adaptation. Furthermore, given the role of the ACC in comparing option values over time (Kolling et al., 2012, 2014), the observed relationship with variance in value from trial to trial could reflect a cognitively meaningful surprise signal, potentially related to environmental volatility (Behrens et al., 2007). Recent work observes just such a signal in a biophysically plausible model of reward learning, in which learning is adapted to volatility via metaplasticity (Farashahi et al., 2017).

To conclude, we find that a distributed network comprising the intraparietal sulcus, inferior frontal gyrus, and posterior cingulate and superior temporal sulcus contribute to the computation of integrated value in the ACC. The strength of signals in the ACC reflected the degree to which they were represented in brain areas coding for quality (IFG) and quantity (IPS), and stronger brain correlations with quantity were associated with more predictable choices. We further demonstrate that parts of the ACC also show repetition suppression to integrated value, which is consistent with the idea that tuning for value is nonmonotonic in parts of the cortex. Our findings demonstrate how value is assembled from its component parts and emphasize the potential for repetition suppression as an assay of a population-encoding scheme.
